# The *hMLH1* −93G>A Polymorphism and Risk of Ovarian Cancer in the Chinese Population

**DOI:** 10.1371/journal.pone.0135822

**Published:** 2015-08-14

**Authors:** Leilei Niu, Shumin Li, Huamao Liang, Hua Li

**Affiliations:** 1 Department of Obstetrics and Gynecology, Peking University Third Hospital, Beijing, China; 2 Department of Gynecology Oncology, Cancer Hospital, Chinese Academy of Medical Sciences, Beijing, China; Medical College of Soochow University, CHINA

## Abstract

**Background:**

As a mismatch repair (MMR) gene, *hMLH1* plays an important role in the maintenance of chromosomal integrity. Several studies have investigated the associations of *hMLH1* -93G>A (rs1800734) and Ile219Val (rs1799977) in diverse tumor types with discordant results, but their roles in ovarian cancer in the Chinese population remains to be elucidated.

**Methods:**

In a case-control analysis, we assessed the association between these two polymorphisms and ovarian cancer risk in 421 ovarian cancer patients and 689 control subjects in the Chinese population using logistic regression.

**Results:**

We found that the variant *hMLH1* genotypes (-93AA and AG) are associated with risk of ovarian cancer (adjusted odds ratio [OR] = 2.02, 95% confidence interval [CI] = 1.42–2.89) compared with the -93GG genotype. The A allele increases the risk of ovarian cancer in a dose-dependent manner (*P*<10^−4^). Functional test showed that -93A allele increased *hMLH1* promoter transcriptional activity and the luciferase activity. However, no significant difference was found in the genotype frequencies at the Ile219Val site between the cases and controls.

**Conclusions:**

These findings indicate that the -93G>A polymorphism in *hMLH1* may affect ovarian cancer susceptibility in the Chinese population.

## Introduction

Ovarian cancer is one of the most common types of gynecological malignancies [1]. Globally, as of 2010, approximately 160,000 people had died from ovarian cancer, up from 113,000 in 1990 [2]. Epidemiological surveys have demonstrated that some etiologic factors, including early age at menarche, late age at menopause, obesity, use of estrogen and hormone-replacement therapy, and inherited susceptibility, are positively associated with ovarian cancer [3, 4]. Additionally, the incidence of ovarian cancer increases with increasing age. Because a standard therapy exists but does not obtain ideal outcomes, ovarian cancer remains a therapeutic challenge. Recent studies indicate that there is significant difference in the clinical outcomes of radical operation and adjuvant platinum-based chemotherapy for individuals with different genetic polymorphisms, suggesting that genetic susceptibility plays an important role in an individual’s risk of developing ovarian cancer [5, 6]

DNA repair systems have an essential role in maintaining genomic integrity and stability. It well known that genetic variants of repair genes may affect the function of the encoded proteins and alter their ability to repair DNA, which in turn may lead to susceptibility to different types of cancer and can also be adverse prognostic factors once cancer has developed [7, 8]. As a key component of the DNA mismatch repair (MMR) system, *hMLH1* (human mutL homolog 1) physically interacts with other components of MMR and plays a crucial role in maintaining genome stability in responses to replication errors and physical or chemical damage to DNA [9]. Several findings have suggested the essential role of *hMLH1* in the control of the cell cycle and apoptosis [10–12]. Dysfunction of the *hMLH1* gene may result in failure to accomplish all the functions of the MMR system and may be associated with a predisposition for cancer [13, 14]. Several polymorphisms have been identified in the *hMLH1* gene [15–17]. Recently, studies have explored the association of an important and frequent polymorphism, namely -93G>A (rs1800734), which is located in the core promoter region of *hMLH1*, with susceptibility to developing various human malignancies, including tobacco-related oral carcinoma [18], lung cancer [19], colorectal cancer [20] and papillary thyroid carcinoma (PTC) [21]. Another widely studied missense polymorphic site in the *hMLH1* gene is Ile219Val, an A to G transition occurring in codon 219 that leads to an amino acid change that has been studied in cancers in European populations [22, 23]. Though the fact that the *hMLH1* gene have been linked to several types of cancer, the association of these variations with ovarian cancer in the Chinese population has not been studied. We hypothesized that the *hMLH1*gene polymorphisms -93G>A and Ile219Val (rs1799977) may cause individual susceptibility to ovarian cancer by affecting the DNA mismatch repair capacity.

Considering the important role of *hMLH1* in the carcinogenic process, we carried out a case-control study in a Chinese population to investigate the possible relationship between these two polymorphisms and the risk of ovarian cancer in the Chinese population.

## Materials and Methods

### Study population

Eligible patients (421) were those diagnosed ovarian cancer consecutively recruited from March 2003 to May 2009 at Peking University Third Hospital (Beijing) and the Cancer Hospital of the Chinese Academy of Medical Sciences (Beijing). The patients were from Beijing city and its surrounding regions under the age of 90 years. The controls (689) were frequency matched to the cases by age (±5 years) lived in the Beijing region and were selected randomly from those who underwent a physical examination in hospitals. The response rate was 96.5%. Physical examination included chest radiography, abdominal ultrasonography, gynecological and cervical cytological examinations. The selection criteria included no history of cancer or previous operations on ovary. The tumor histological status and clinical staging were based on the criteria of the International Federation of Gynecology and Obstetrics (FIGO) [24]. The clinical features of the patients for our study are presented in **Table 1**. At recruitment, all participants were asked to provide written informed consent for collecting blood of them and completed a structured questionnaire. The study procedure was approved by Peking University Third Hospital and the Cancer Hospital of the Chinese Academy of Medical Sciences.

**Table 1:** Clinical characteristics of ovarian cancer patients and healthy control subjects.

Characteristics	Cases (421)		Controls (689)	
	N	(%)	N	(%)
**Age (years)**				
≤40	58	(13.8)	81	(11.8)
41–60	261	(62.0)	457	(66.3)
≥60	102	(24.2)	151	(21.9)
**Body mass index (BMI)**				
≤20	116	(27.6)	171	(24.8)
20–28	273	(64.8)	462	(67.1)
≥28	32	(7.6)	56	(8.1)
**Pathological type**				
Serous-papillary	166	(39.4)		
Mucinous	54	(12.9)		
Endometrioid	150	(35.6)		
Undifferentiated	51	(12.1)		
**FIGO stage**				
I	61	(14.5)		
II	80	(19.0)		
III	226	(53.7)		
IV	54	(12.8)		
**Tumor grade**				
G1	36	(8.6)		
G2	99	(23.5)		
G3	286	(67.9)		

FIGO: International Federation of Gynecology and Obstetrics

### Genotyping analysis

Genomic DNA was isolated from the peripheral blood of the study subjects using the DNA Blood Mini kit (QIAGEN, Valencia, CA). MALDI-TOF platform were used to genotype *hMLH1* -93G>A and Ile219Val polymorphisms as described previously [25, 26]. For quality control, 5% samples were randomly selected to repeat, and 50 samples were included for direct sequence to confirm genotypes from the mass spectrometric analysis. Hardy-Weinberg was checked in all the sample groups.

### Reporter plasmids, transfections and luciferase Assays

The 93G and 93A allelic reporter constructs were prepared by the Genewiz Company (Beijing, China) by amplifying the core promoter region of *hMSH1*, a 282-bp fragment from -299 to -17, as previously described [27], and then cloned into the pGL3-basic luciferase vector (Promega, Madison, WI, USA). 293T cells and SKOV-3 cells (ovarian cancer cell line) were used to transfect the above reporter plasmids with Lipofectamine 2000 (Invitrogen, Carlsbad, CA, USA) through previously published procedures [28, 29], the empty vector was used as a negative control. The Renilla luciferase activities were measured with the Dual-Luciferase Reporter assay system (Promega).

### Real-Time Analysis of *hMLH1* mRNA

The total RNA from twenty-eight ovarian cancer tissue samples obtained from biopsy-removed specimens of individual patients was extracted using the TRIzol reagent (Invitrogen, Inc.). The relative gene expression quantification of *hMLH1* was conducted using the SYBR Green Assay using the ABI Prism 7500 sequence detection system (Applied Biosystems). β-actin was used as endogenous controls for *hMLH1* expressions. The primers used for *hMLH1* were 5’-CAGAGGAAGATGGTCCCAAA-3’ (F) and 5’-TCTTCGTCCCAATTCACCTC-3’ (R), and those for β-actin were 5’-GGCGGCACCACCATGTACCCT-3’ (F) and 5’-AGGGGCCGGACTCGTCATACT-3’ (R). The relative expression of *hMLH1*was performed by using the 2^-ΔΔCT^ calculation.

### Statistical analysis

Two-sided chi-square tests were used to assess differences in the age and body mass index (BMI) distributions as well as the allele and genotype frequencies between the cases and controls. The observed genotype frequencies in the cancer-free controls was tested for Hardy–Weinberg equilibrium (HWE) by goodness-of-fit *x* ^2^ tests. The associations of all genotypes with risk of ovarian cancer were calculated by odds ratios (OR) and corresponding 95% confidence intervals (CIs) followed by stratification analysis with adjustment for age and BMI. The statistical power was computed by applying the PS software (http://biostat.mc.vanderbilt.edu/twiki/bin/view/Main/PowerSampleSize, accessed Dec 14, 2010). The differences in the luciferase reporter activity and mRNA levels of *hMLH1* in ovarian cancer tissues between different each allele were examined by one-way ANOVA and Student’s t test. The LD of the two SNPs was detected by the 2LD program and the PROC ALLELE statistical procedure in SAS/Genetics (SAS Institute, Inc., Cary, NC, USA) software. *P*<0.05 was used as the criterion for statistical significance, and all tests were two-sided and performed with the SAS software (version 9.1; SAS Institute, Cary, NC, USA).

## Results

### Genotypes and risk of ovarian cancer

The association of ovarian cancer with -93G>A was determined based on 421 cases and 689 controls. The genotyping results in **Table 2**showed that *hMLH1* -93G>A was significantly associated with ovarian cancer risk. Compared to the healthy controls, -93AA and AG carriers were significantly associated with risk of ovarian cancer (adjusted odds ratio [OR] = 2.02, 95% confidence interval [CI] = 1.42–2.89). Additionally, the -93A allele frequency in cases (65.6%) was also significantly higher than that in the controls (52.4%) (*P*<10^−4^). However, there was no significant difference between the different genotypes of the variant Ile219Val and susceptibility to ovarian cancer. Linkage disequilibrium analysis showed that the linkage between -93G>A and Ile219Val was relatively weak (r^2^ = 0.051), suggesting that each may have an independent effect on the risk of ovarian cancer and therefore be unsuitable for haplotype analysis.

**Table 2:** Genotype freqencies of the two SNPs in *hMLH1* among patients and controls and their associations with ovarian cancer.

Genotypes	Controls (689)		Cases (421)			
	N^a^	(%)	N^a^	(%)	OR (95%CI)^b^	*P*_trend_
**−93G>A**						
GG	150	(21.8)	51	(12.1)	1.00 (Reference)	
AG	356	(51.7)	188	(44.7)	1.54 (1.05–2.27)	<10^−4^
AA	183	(26.5)	182	(43.2)	2.92 (1.96–4.34)	
AG+AA	539	(78.2)	370	(87.9)	2.02 (1.42–2.89)	
G	656	(47.6)	290	(34.4)	1.00 (Reference)	
A	722	(52.4)	552	(65.6)	1.73 (1.44–2.06)	
**Ile219Val**						
AA	613	(89.0)	386	(91.7)	1.00 (Reference)	
AG	75	(10.9)	33	(7.8)	0.70 (0.43–1.08)	0.217
GG	1	(0.1)	2	(0.5)	/	
AG+GG	76	(11.0)	35	(8.3)	0.73 (0.48–1.13)	
A	1301	(94.4)	805	(95.6)	1.00 (Reference)	
G	77	(5.6)	37	(4.4)	0.78 (0.51–1.17)	

^a^Number of cases/number of controls.

^b^Data were calculated by logistic regression analysis with adjusted for age and BMI.

The risk of ovarian cancer related to the *hMLH1* -93G>A genotypes was further determined with stratification by age and BMI. No significant association between the variant genotypes and risk of ovarian cancer was observed.

### Effects of the *hMLH1* -93G>A polymorphism on transcriptional activity

**Fig 1:**
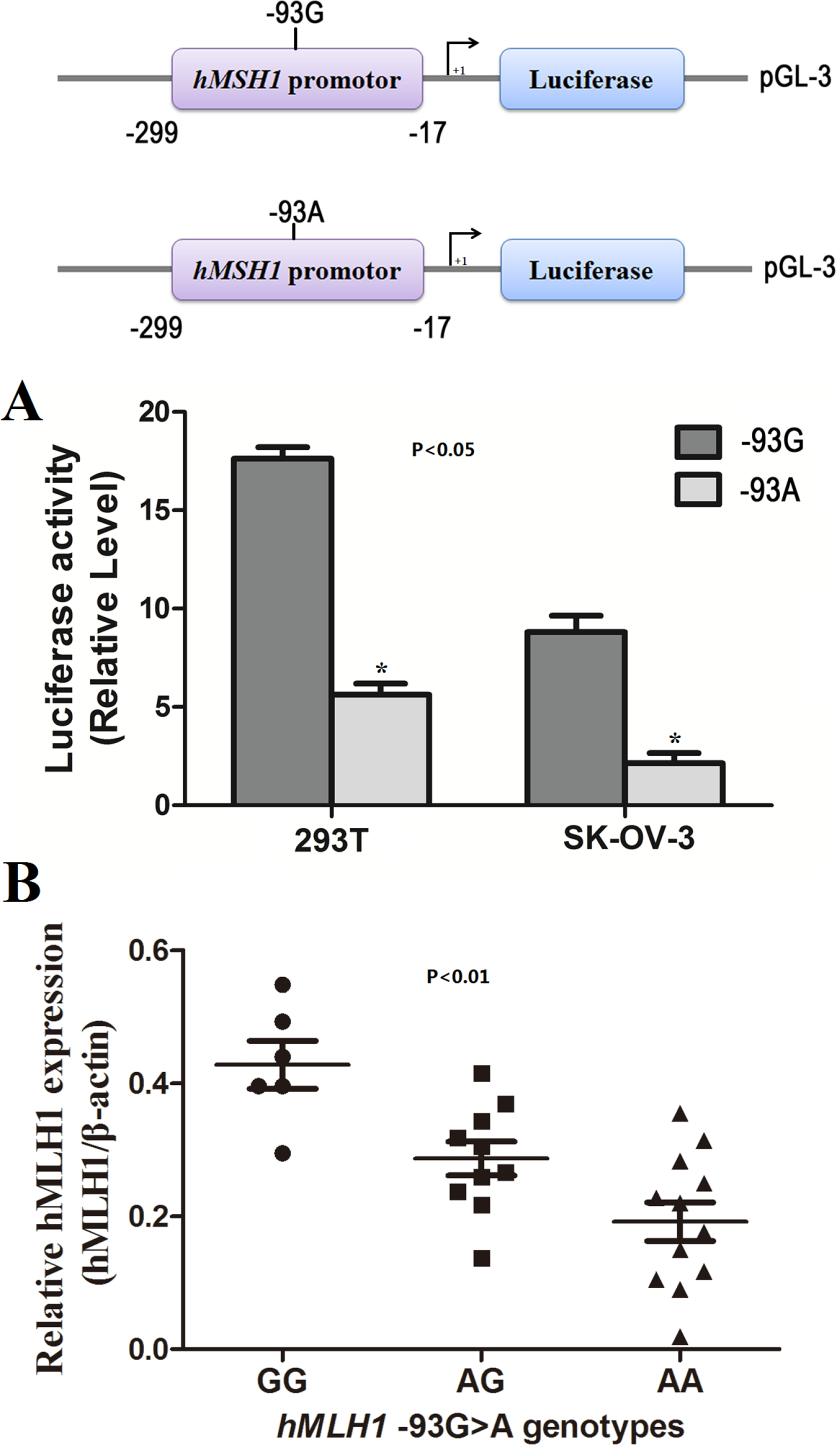
The effect of the *hMLH1* -93G>A genotype on *hMLH1* expression. **(A)** Schematic of reporter gene constructs containing the core promoter region of *hMSH1* (a 282-bp fragment from -299 to -17) with the G or A allele at the -93 polymorphic site (upper). The luciferase expression of two constructs (pGL3-93G and pGL3-93A) in 293T and SK-OV-3 cells cotransfected with pRL-SV40 to standardize the transfection efficiency is shown (lower). Each group has six replicates, and the transfection experiments were repeated three times. The data are the means ± standard error of the mean. The asterisk indicates a significant change. (**B**) *hMLH1* expression level in twenty-eight ovarian cancer tissues with different -93G>A genotypes (6 -93GG, 10 -93AG and 12 -93AA); the data are the means ± standard error of the mean. The expression level of the -93GG and -93AG genotypes were significantly higher than that of the AA genotype (*P*<0.001).

Considering that -93G>A polymorphism could be associated with risk of ovarian cancer, two luciferase reporter constructs (pGL3-*hMLH1*-G-allele and pGL3-*hMLH1*-A-allele) were used to transiently transfect 293T cells to directly determine the effects of the -93G>A polymorphism on the transcriptional activity of the *hMLH1* promoter. As shown in **Fig 1A**, compared with the construct containing the -93G allele, the construct containing the -93A allele exhibited significantly higher luciferase activity (*P*<0.001). Consistent with increased *hMLH1* promoter transcriptional activity for the -93A allele in the 293T cells, the luciferase activity of the construct containing the -93A allele appeared to be higher in cell lines in SK-OV-3 cells (*P* = 0.002).

### Association of *hMLH1* -93G>A genotypes with *hMLH1* expression

To further evaluate the effect of SNP -93G>A on ovarian cancer risk, we examined *hMLH1* expression between different genotypes of the SNP in twenty-eight individual ovarian cancer tissues (6 -93GG, 10 -93AG and 12 -93AA) by real-time PCR. As shown in **Fig 1B**, in the twenty-eight ovarian cancer tissues, *hMLH1* expression was significantly higher in those with the -93GG and -93AG genotypes than in those with the -93AA genotype (GG, 0.428±0.036; AG, 0.287±0.026; AA, 0.192±0.029; ANOVA test: *P*<0.001).

## Discussion

In the present hospital-based case-control study, we sought to identify genetic factors that confer individual susceptibility to ovarian cancer. The results obtained by analyzing 421 patients and 689 healthy controls showed that *hMLH1* -93G>A is associated with risk for ovarian cancer in the Chinese population. Our data showed that, compared with the GG genotype, subjects carrying the -93AA and AG genotypes had significantly increased risk for ovarian cancer (*P*<10^−4^).

Recently, the mutation of DNA repair genes in the etiology of several cancers has been receiving a great deal of attention. It has been proposed that genetic variants in DNA repair genes that potentially result in altered protein expression or function would lead to failure in DNA damage recognition and repair, which in turn would allow subsequent mutations to accumulate and thus might increase susceptibility to multiple cancers [30–32]. In humans, there are several DNA repair pathways that are essential for genome integrity, including base excision repair (BER), nucleotide excision repair (NER), DNA double-strand breaks (DSBs) and MMR [33]. A highly conserved set of MMR proteins has long been known to be primarily responsible for repairing bases mismatched during DNA replication [34, 35]. However, recent studies have commonly reported that *hMLH1* plays a central role in the MMR system in mismatch strand excision and subsequent repair [36, 37]. The genetic variant of the *MLH1* gene may affect the MMR capacity of the encoded protein and therefore might contribute to the risk of cancer. To date, there has been a great deal of interest in the association between two widely studied polymorphisms (-93G>A and Ile219Val) and different types of cancer; however, the results are contradictory. The common polymorphism *hMLH1* -93G>A is located in the core promoter region, 93 nucleotides upstream of the transcription start site, contains putative consensus sequences for transcription factor binding sites, and may play an important role in regulating *hMLH1* promoter activity, thereby modulating susceptibility to cancer [38]. The *hMLH1* -93G>A polymorphism has been investigated in several studies, but the results are not consistent. Recently, *hMLH1* -93G>A variant genotypes (AA, AG) have been associated with an increased risk of lung cancer [39], breast cancer [40] and colorectal cancer [41]. Interestingly, a recent study of Asian Indians using 242 oral carcinoma patients and 205 healthy controls found that the GG genotype showed significantly higher prevalence in patients compared to the healthy controls (P<0.0006) [18]. Our results indicated that the variant genotypes (AA, AG) of *hMLH1* -93G>A may be related to cancer risk.

Another commonly studied *hMLH1* polymorphism, Ile219Val, is a non-synonymous mutation that changes Ile 219 to Val, possibly altering the function of the hMLH1 protein. Several studies have reported that the *hMLH1* Ile219Val polymorphism is closely related to the onset of a variety of cancers, including lung cancer [42], breast cancer [40] and gastric cancer [43]. However, the association between *hMLH1* Ile219Val variant genotypes and cancer risk is not the same in different cancers and ethnic groups. For example, in prostate cancer, Burmester et al. found that the *hMLH1* Ile219Val polymorphism is significantly associated with higher rates of prostate cancer in people of European ancestry, whereas Fredriksson et al. showed that *hMLH1* did not have a major role in prostate cancer in a Finnish population [22]. Our results suggested that there is no association between this polymorphism and ovarian cancer in the Chinese population.

Certain limitations may exist in the present study because of its hospital-based design limited to Chinese population. Additionally, inherent selection bias that introduces unbalanced confounding factors might affect the conclusions of the current our results, because cases and controls were from different centers. However, the genotype frequencies among the controls fit the Hardy-Weinberg disequilibrium law, suggesting random subject selection, and we achieved a more than 90% study power (two-sided test, α = 0.05) to detect an OR of 2.02 for the *hMLH1*–93 AA+AG genotypes (which occurred at a frequency of 78.2% in the controls) compared with the -93GG genotype, suggesting that this finding is noteworthy.

In conclusion, the present study indicates that in the Chinese population, carriers of the -93AA and AG genotypes have increased risk of ovarian cancer compared with GG carriers. To the best of our knowledge, this study provided the first evidence that the -93G>A polymorphism in *hMLH1* is associated with a significant risk of developing ovarian cancer in the Chinese population. Further independent population-based case-control studies are warranted to validate our results in larger sample sizes, as well as in different populations.
