# Correction: The *hMLH1* −93G>A Polymorphism and Risk of Ovarian Cancer in the Chinese Population

**DOI:** 10.1371/journal.pone.0218032

**Published:** 2019-05-31

**Authors:** Leilei Niu, Shumin Li, Huamao Liang, Hua Li

After publication of this article [1], a reader noted that the description of Fig 1A findings in the Results section does not agree with the results reported in the figure. The authors clarified that there are errors in the Results section text such that allele references are incorrect. In the “Effects of the *hMLH1* -93G>A polymorphism on transcriptional activity” subsection of the Results, the second and third sentences should read (corrected text is in bold):

“As shown in Fig 1A, compared with the construct containing the **-93A** allele, the construct containing the **-93G** allele exhibited significantly higher luciferase activity (P<0.001). Consistent with increased *hMLH1* promoter transcriptional activity for the **-93G** allele in the 293T cells, the luciferase activity of the construct containing the **-93G** allele appeared to be higher in cell lines in SK-OV-3 cells (*P* = 0.002).”

In addition, the primary data underlying the results were not included in the published article although the Data Availability statement reads, “All relevant data are within the paper.” The authors uploaded underlying data for Fig 1 to the Open Science Framework (https://osf.io/86zre/). The Fig 1A legend says, ‘Each group has six replicates, and the transfection experiments were repeated three times.’ The data file posted to the Open Science Framework includes only three luciferase activity values for each experimental condition. The authors provide the following clarifications about these data:

For Fig 1A, the values provided in the Excel file are the data from which Fig 1A was drawn. So it is the summary data for three independent experiments. Now, we have provided the raw data of each individual experimental values at Open Science Framework. The number of experimental replicates reported in the paper is correct. The relative firefly luciferase activities were normalized against the Renilla luciferase activities. The data are the means ± standard error of the mean from three independent experiments.

For Fig 1B, the columns (A and B) represent the CT values of β-actin used as an endogenous control and *hMLH1*, respectively. The column C represents the difference value of *hMLH1* (CT) and reference (CT). The column D represents the relative gene expression data of *hMLH1* using the 2^-ΔΔCT^ calculation method. The column E represents the genotypes of the SNP −93G>A in twenty-eight individual ovarian cancer tissues. The data are presented as means ± standard error.

The underlying data for clinical results presented in Tables 1 and 2 are attached here in a Supporting Information file.

The authors apologize for the errors in the published article.

## Supporting information

S1 File Data for Tables 1 and 2. The underlying data for clinical results presented in Tables 1 and 2.
(XLSX)
